# Diffraction perception in L-shaped rooms using virtual reality

**DOI:** 10.1186/s13636-025-00433-2

**Published:** 2025-12-20

**Authors:** Joshua Mannall, Annika Neidhardt, Paul Calamia, Lauri Savioja, Russell Mason, Enzo De Sena

**Affiliations:** 1 Institute of Sound Recording, University of Surrey, Guildford, UK; 2 Sound Inference, LLC, Spokane, USA; 3 Department of Computer Science, Aalto University, Espoo, Finland

**Keywords:** Diffraction, Virtual reality, Perception

## Abstract

Outside of shoebox rooms, acoustic diffraction phenomena are present and can influence important aspects of auditory perception, such as localisation. A simple extension of a shoebox room is an L-shaped room as it introduces a single diffracting edge. This paper presents two experiments carried out in L-shaped rooms in virtual reality. The first investigated whether the inclusion of diffraction modelling influences the perceived plausibility of the acoustic simulation, and the second to what extent newly developed efficient IIR filter diffraction models are equally plausible to the physically accurate Biot-Tolstoy-Medwin-Svensson (BTMS) model. The study compared diffraction of only the direct sound and diffraction of both direct and reflected sound. The results show that the inclusion of diffraction increased the perceived plausibility of the acoustic simulation. A statistically significant increase in plausibility was found by the addition of diffracted reflection paths, but only in the so-called shadow zone. The second experiment determined that the IIR filter diffraction models were similarly plausible to BTMS in 14 of 18 cases with a threshold of 0.5 on a 6-point Likert scale.

## Introduction

Creating accurate acoustic simulations has been shown to improve users’ sense of immersion, presence [1–3], and externalisation [4, 5] within virtual environments. However, since computing resources for audio are often limited in these applications, computationally efficient models are required.

One of the current challenges is efficient diffraction modelling, in which the interaction of sound with an edge is accounted for. Perceptual experiments have shown that diffraction modelling causes audible changes in the sound field [6, 7] and that accurate diffraction modelling leads to improvements in subjective measures [8, 9]. In wave-based simulations such as finite-difference time-domain [10] and adaptive rectangular decomposition [11, 12] diffraction phenomena are inherently included by solving the wave equation. These models are usually precomputed for a known geometry, which makes implementing dynamic scenarios, such as the change of acoustics when a door opens or closes, challenging. Alternatively, many real-time simulations use the class of geometrical acoustics (GA) models, which have significantly lower computational complexities [13–15]. Here, the propagation of sound is approximated by multiple rays emitted from a source. This approximation assumes that the wavelength of sound is significantly smaller than the geometric features within the acoustic scene [14]. Incorporating diffraction modelling as part of a GA model can improve accuracy, especially at low frequencies [16].

Modelling edge diffraction as part of a GA model involves path finding, i.e., locating diffraction paths [15, 17], and rendering, i.e., applying a frequency-dependent response based on geometric parameters [18–21]. Both of these can be considered when looking for efficiency gains. The computational complexity of path finding can be reduced through the development of fast path finding algorithms [15]. A complementary approach is to decrease the number of diffraction paths included in the simulation. In this case, it is important to understand the perception of diffraction as part of a complete room acoustic model in order to maintain an acceptable level of perceivable accuracy and immersion. Secondly, when rendering diffraction paths, efficiency gains can be created by developing an efficient model and filter structure.

The current literature does not consider how reverberation might change the perception of diffraction, and to the best of the authors’ knowledge, perceptual evaluations of the most recent efficient diffraction models have not yet been carried out. Therefore, this paper presents two experiments. The first, previously presented in [22], aims to investigate how reverberant energy (RE) impacts the perceptual relevance of diffraction modelling in virtual reality (VR) and how this changes with the listener’s position relative to the edge. The experiment also investigates the perceptual relevance of applying diffraction to reflection paths, as previous work in this area [6] was inconclusive. The physically accurate Biot-Tolstoy-Medwin-Svensson (BTMS) model [18] is used as the diffraction model. The results of this experiment are used to run a second experiment in scenarios where diffraction is known to increase the plausibility of an acoustic simulation. It aims to investigate whether efficient IIR filter models [15, 20, 21] can replace the BTMS model [18] without reducing plausibility.

The paper is structured as follows. Section 2 outlines previous research on diffraction rendering and its perception. Section 3 presents the design of the VR simulation and experiment methodology. The results of each experiment are presented and discussed in Sects. 4 and 5. Section 6 concludes the paper.

## Background

### Edge diffraction models

**Fig. 1:**
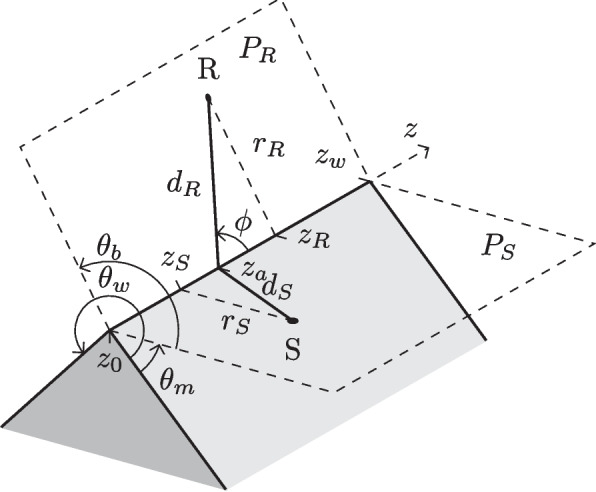
Edge geometry described using cylindrical coordinates. S denotes the sound source and R the receiver. Adapted from [23]

Geometrical acoustics are used to describe the propagation of sound as rays, but neglect wave phenomena such as diffraction. Many diffraction models introduce diffracting rays that connect the shortest path between a source and receiver via a diffracting edge. This diffraction path can be given as a set of geometric parameters describing the relative positioning of the source, receiver and diffracting edge, shown in Fig. 1.

**Fig. 2:**
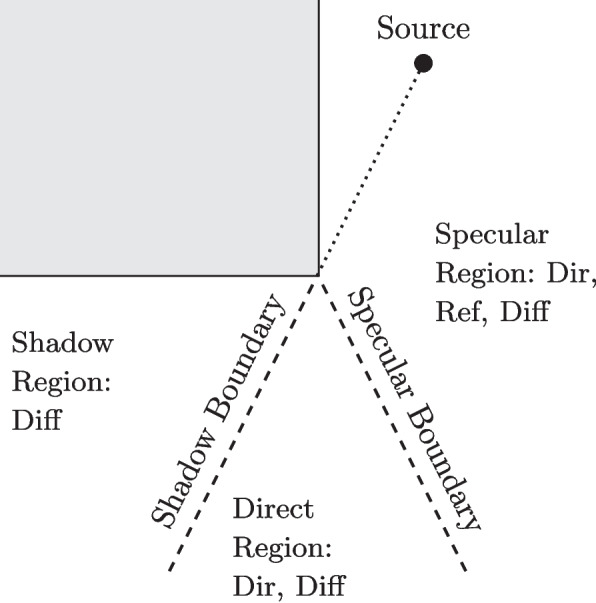
Regions around an edge showing where direct (Dir), reflected (Ref) and diffracted (Diff) sound components are present. Adapted from [23]

The space around a diffracting edge can be divided into three regions with two important boundaries, as shown in Fig. 2. The specular boundary divides the regions where the specular reflection is or is not, and the shadow boundary corresponds to where the direct sound component changes from visible to non-visible. Several models have been proposed to calculate the frequency response of a given diffraction path from the parameters in Fig. 1, the most well known in the literature are the uniform theory of diffraction (UTD) [19] and BTMS [18]. Recently, more efficient models: NN-IIR [21] and Universal Diffraction Filter Approximation (UDFA) [20, 24], have been proposed.

Keller’s geometrical theory of diffraction (GTD) [25], originating from the field of geometrical optics, determines a diffraction coefficient based on the wedge angle and relative positions of the source and receiver. The UTD [19] model solved singularities in GTD that appeared in close proximity to the shadow boundary and made the theory suitable for acoustic applications. As it derives from geometrical optics, this model assumes that individual components of the scene geometry are significantly larger than the wavelength of the sound and considers all edges to be infinitely long. Therefore, it exhibits significant errors at low frequencies when the diffracting edge is small relative to the wavelength. It can be processed using convolution or approximated using infinite impulse response (IIR) filters [15].

In 1957, Biot and Tolstoy [26] proposed a model for diffraction by infinite edges. This was then extended by Medwin [27, 28] as a time-domain approach for diffraction by finite edges. BTMS is an analytical solution proposed by Svensson et al. [18] and is widely used in the literature, as it has been shown to closely match measurements [29, 30]. The model divides a single edge into multiple edge sources, and by applying Huygens’ principle of secondary sources, it is possible to calculate the time-domain response of the diffracting edge through an integration over the length of the edge. The result can be applied as an finite impulse response (FIR) filter or through overlap-add convolution.

Recently NN-IIR [21] was proposed as a machine learning approach to the problem of edge diffraction. The model, trained using BTMS, predicts the poles, zeros and gain of a 2nd-order IIR filter based on input geometrical parameters. Kirsch et al. [20, 24] proposed UDFA which derives fractional filter approximations for diffraction by an infinite edge. Since finite edges are truncated responses of an infinite edge, an approximation can be made by adjusting the DC gain and cut-off frequency of the filter based on the integral along the diffracting edge. For real-time applications, they fit serially arranged 1st-order IIR shelving filters to the fractional filter responses.

### Perception of diffraction

Torres et al. [6, 16, 31, 32] wrote a series of papers aiming to determine a threshold of perception for diffraction modelling. They found that (a) 1st-order diffraction was audible, (b) diffraction was more audible with low-frequency and wide-band programme material and (c) diffracted reflection paths may or may not be audible. Listening experiments by Calamia et al. [7] found that modelling a small number of diffraction paths can be difficult to distinguish from a simulation that includes all possible diffraction paths. They selected the most significant paths on the basis of amplitude. In a second experiment, they proposed using the angular distance from the shadow or reflection boundary and the apex point (where the diffraction path intersects the edge) to cull paths as these are indicators for when the amplitude of diffraction will be greatest. They noted that this method became less effective with occluded sources and that high amplitudes due to short distances between the source and receiver were not taken into account.

Other recent work has conducted perceptual experiments that compare diffraction models [8, 9]. Rungta et al. [8] compared the GA-based UTD model with the wave-based adaptive rectangular decomposition (ARD) model. This found that the perceived decay in volume as a listener moved around an obstacle was strongly linear for ARD, but UTD deviated from the expected linear decay. In a comparison of multiple GA-based diffraction models, Mannall et al. [9] concluded that improving frequency response accuracy increased the perceived naturalness of a model.

An important aspect of diffraction perception is its effect on the perceived location of a sound source. This has been investigated for anechoic conditions in [33, 34]. These studies found that the diffraction of sound around an occluding baffle shifts the perceived location of the sound source towards the nearest diffracting edge. The same phenomenon was found in experiments using a GA model. Other studies have investigated whether acoustic cues from diffraction help players navigate virtual environments. Cowan et al. [35] found that navigation speed and accuracy increased when diffraction cues were correctly modelled and that this was most significant for participants with less gaming experience. They noted that participants had a higher tendency to wander off course when the acoustic cues were less accurate and that experienced gamers had less trust in the acoustic cues and tended to perform visual sweeps of an area before moving on. A more recent study by Garí et al. [36] found no significant differences in navigation times when diffraction modelling was included in the simulation. These contrasting results may be due to differences in the scenes. The first study included a complex indoor and outdoor environment with multiple floors, while the second study was an indoor maze-like environment with high levels of reverberation that may have masked the diffraction. Secondly, the sources in the second experiment were invisible, which may have made the navigation task more challenging for participants, as some participants marked the location of sources incorrectly, for example behind walls.

### Summary

Previous experiments have shown that diffraction modelling causes audible changes in the sound field [6, 7] and that accurate diffraction modelling leads to improvements in subjective measures [8, 9]. There have been limited comparisons between diffraction models and the relationship between reverberation and diffraction perception has not been explored.

## Experiment framework

**Fig. 3:**
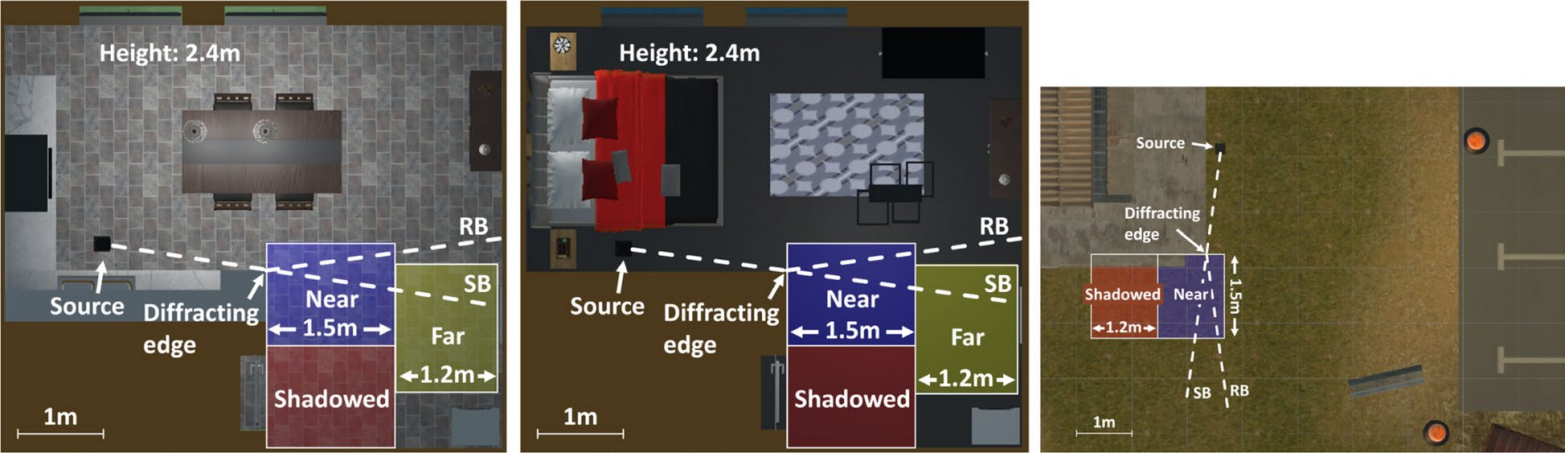
The three scenes showing the three zones used in the listening experiment. The source positions and main diffraction edges are labelled. The dashed lines show the reflection boundary (RB) and the shadow boundary (SB)

This section describes two listening experiments investigating how diffraction is perceived in a virtual environment. Paired comparisons were made between different acoustic rendering methods, simulations with and without physically accurate diffraction modelling (Experiment 1) and using different diffraction models (Experiment 2) in two L-shaped rooms with differing reverberant energy (RE), an outdoor scene and in three zones around a diffracting edge, shown in Fig. 3.

Two methodologies can be considered useful for assessing the perceptual importance of diffraction within an acoustic model. The first is to test the perceived similarity between stimuli with and without diffraction modelling. If diffraction is inaudible, it can be discounted from the acoustic simulation, however, this does not provide information on whether the reference simulation is correct. In VR there is no real reference to compare with, so while the acoustic model is physically motivated, it is impossible to guarantee true authenticity. The second measure is plausibility. Lindau [1] defines plausibility as the “agreement with the listener’s expectation towards a corresponding real event”. This is a useful measure for assessing a model in VR as instead of a real reference; listeners must rely on their internal reference and assess how convincing the acoustic simulation is given the visual representation of the room. For this experiment, participants were asked to rate both similarity and plausibility. Two stimuli can be equally plausible or implausible but dissimilar and this methodology allows this information to be elicited from subjects.

### Acoustic model

The RoomAcoustiC++ (RAC)^1^ [37] implementation was used as the acoustic model in the listening experiment. It is a hybrid GA-FDN model [38] whereby an image edge (IE) model [17] simulates early reflections and diffraction and the highest order image sources are used to feed the input of a feedback delay network (FDN) that models late reverberation. The maximum IE order determines the maximum number of combined reflections and diffractions along an image source (IS) path. This was set to three, as, at higher orders, most of the reflected sound energy is diffuse [39]. The sample rate was set to 48 kHz.

The FDN parameters were set as 12 input and output channels, a random orthogonal feedback matrix and reverberation time (RT) estimated using the Eyring equation [40]. In RAC, the FDN delay line lengths are determined by room dimensions that are manually defined by the user. In an L-shaped room, there are two main distances (one long, one short) in each of the two horizontal dimensions. Each of these four distances were applied to two delay lines. The vertical dimension was applied to the remaining four delay lines.

The material absorption coefficients were defined in octave bands with center frequencies from 250 Hz to 4 kHz. The filter design in RAC sets the target for frequencies outside this range as the average of the top or bottom two frequency bands. Absorption was defined by assigning coefficients from absorption databases with matching visual materials in the virtual scene. Following an informal listening test, small changes were made (for example, changing from a heavy to a thin carpet) to better correlate RT with the visual scene. This is a challenging task as expectations for reverberation based on the visual impression of a room vary greatly between listeners [41].

The head related transfer function (HRTF) processing used the KEMAR head HRTF dataset from [42]. RAC uses the 3D Tune-In toolbox [43] for binaural spatialisation. This samples the HRTF in defined angular increments (5-degree resampling was used) and uses barycentric interpolation to interpolate between these sampled directions. Therefore, the HRTF supplied must be temporally aligned with stored interaural time difference (ITD) delays to avoid audible artifacts. To achieve this, the Matlab function rceps was used to convert the HRTF to minimum phase. The ITDs were estimated using first-onset threshold detection with a 3 kHz low-pass filter and −30 dB threshold, as recommended in [44].

### Virtual environment

Measuring plausibility is based on a subject’s internal reference of how a given space should sound [45]. Therefore, it is important that the scenarios presented to participants represent relatable environments [9]. The three test scenes consisted of an outdoor area (experiment 2 only), a bedroom and a kitchen diner. The two rooms were similar in size and shape, with only slight changes due to differing furniture. When designing the scenes, care was taken to ensure that the scale and material textures were realistic, as these provide important visual cues about distance [46]. The material properties of the two spaces lead to differing RTs and REs. The RTs in octave bands from 250 Hz to 4 kHz were: bedroom (0.46s, 0.38 s, 0.30 s, 0.27 s, 0.25 s) and kitchen diner (0.60 s, 0.50 s, 0.47 s, 0.46 s, 0.44 s). No late reverberation was included in the outdoor scene. For the remainder of this paper, RE is defined as the total energy in dB of the room impulse response, excluding direct sound. A discussion of how RE changes between scenes is presented in Sect. 4.4.

Three zones were created to examine how plausibility changed based on the listener’s position in relation to the primary diffracting edge. These are shown in Fig. 3 and were termed: (1) the “Near Zone,” across the shadow boundary at 0 m to 1.5 m from the edge, (2) the “Far Zone,” across the shadow boundary at 1.5 m to 2.7 m from the edge, and (3) the “Shadowed Zone,” situated within the shadow region where the bending angle (see $\theta _b$ in Fig. 1) exceeds $210^{\circ }$. These zones were selected to investigate whether perception changed as the listener transitioned into the shadow region and as their distance from the diffracting edge increased. The dimensions of the zones were chosen such that, considering the room size constraints, the area of each zone was the same. Throughout the test, the active zone was shown visually in the VR scene. If participants left this area, the audio was silenced, and visual instructions were given to return to the highlighted zone.

### Experiment methodology

For the experiments, a pairwise comparison methodology was selected. A Multiple Stimuli with Hidden Reference and Anchor (MUSHRA) test design was deemed unsuitable as the differences between stimuli were sometimes small, making comparisons between multiple stimuli taxing for participants [47]. Two items of programme material were used: female speech (English) and pop music (ABBA), both from the EBU database [48]. During the test, participants were shown pairs of stimuli, where a stimuli represents a given room, zone, programme material and acoustic rendering method. Within each pair, the acoustic rendering method was varied. Each experiment was carried out over two sessions to reduce listener fatigue, with sessions taking between 30 and 45 min.

The test consisted of two familiarisation phases followed by a rating phase. In the first familiarisation phase, participants were shown one stimuli for each of the acoustic rendering methods. The order of these methods was randomised, and for each, the room and programme material were randomly selected. During this phase, participants were not restricted to a single zone and instead were able to navigate within a 4.8m $\times$ 2.2m space that included the areas covered by the three zones. They were instructed to explore the scene and observe how the acoustics changed as they moved. In the second familiarisation phase, subjects were shown three pre-selected pages from the experiment. Each page presented a pair of stimuli with different acoustic rendering methods. These three pages were selected to be representative of the range of differences between stimuli. This phase allowed participants to learn the test controls and the rating scales.

**Fig. 4:**
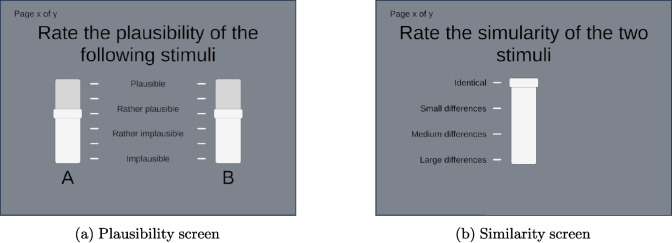
The rating screens shown to participants during the experiment

During the rating phase, participants evaluated pairs of stimuli. First, they were required to rate the *plausibility* of both stimuli using two sliders with discrete six-point scales corresponding to 0–5, as shown in Fig. 4a. Text indicators were included to clarify the meaning of the rating scales. Participants were allowed to switch between the two stimuli freely. On a second page, shown in Fig. 4b, they were asked to rate the *similarity* of the stimuli on a four-point scale corresponding to 0–3. This two-stage elicitation strategy allowed subjects to report stimuli that were audibly different but perceived as equally plausible. The order of the stimuli pairs was randomised and each stimulus within the pairs was randomly assigned to either the A or B slider. The experiment methodology was approved by the University Ethics Committee (UEC) at the University of Surrey (Reference Number: 0934)

### Experimental setup

The experiments were carried out in an ITU-R BS.1116 standard-compliant room with internal dimensions of 7.35 m length, 5.7 m width, and 2.5 m height. Subjects could move within an area of 4.8 m $\times$ 2.2 m.

The simulations were run in Unity on a PC Specialist Flux 310 gaming PC with 16GB RAM, an Intel Core i7-12700F CPU, and an RTX 4060 Ti 8GB graphics card. A Meta Quest Pro headset was connected via a cable using Meta Quest Link. The audio was presented over Pioneer HRM-7 headphones, which could fit comfortably over the band of the headset. The headphones were connected directly to the computer instead of transmitting audio over the Meta Quest Link connection. A pulley system was used to suspend the connecting cables, allowing participants to explore the virtual scenes with six degrees of freedom.

## Experiment 1: plausibility

This experiment aims to determine whether diffraction modelling increases the plausibility of acoustic simulations. The effect of the factors RE, RT and receiver position relative to the diffracting edge on the change in plausibility is investigated.

### Methodology

Table 1 presents the acoustic simulations compared during the test.

**Table 1:** The acoustic simulations compared in Experiment 1: plausibility

Model	Direct	Reflections	Diffraction	Diffracted reflections	Late reverberation
**No Diff**	✓	up to 3rd-order	✗	✗	✓
**Diff**	✓	up to 3rd-order	1st-order	✗	✓
**Refl Diff**	✓	up to 3rd-order	1st-order	Shadow region only	✓
**Anchor**	Not blocked by the room geometry	up to 3rd-order	✗	✗	✓

The physically accurate BTMS model was used for diffraction modelling. Diffracted reflection describes early reflection paths that also include a 1st-order edge diffraction. The anchor was chosen as an example of an implausible stimulus without degrading the quality of the audio signal. The acoustic simulation included approximations for the furniture, and therefore, edges of the bed, table and other cupboards created valid diffraction paths. Due to computational constraints, only diffracting edges longer than 60 cm were allowed to create valid ISs. This reduced the number of ISs in the Refl Diff case and avoided audio buffer underruns. An informal listening test that compared the cases with and without this approximation did not reveal audible differences.

The experiment consisted of two scenes, three zones, two pieces of program material and six comparisons (72 pages). Twelve random pages, shown in Table 2, were selected to be repeated, leading to a total of 84 pages.

**Table 2:** The repeated pages in Experiment 1: plausibility

Scene	Zone	Audio	Comparison
Bedroom	Far	Speech	Anchor/No Diff
Kitchen diner	Shadow	Speech	Anchor/No Diff
Kitchen diner	Near	Music	No Diff/Diff
Bedroom	Near	Music	No Diff/Diff
Bedroom	Shadow	Speech	Diff/Refl Diff
Kitchen diner	Near	Music	Diff/Refl Diff
Kitchen diner	Far	Speech	Anchor/Refl Diff
Bedroom	Shadow	Music	Anchor/Refl Diff
Kitchen diner	Shadow	Music	No Diff/Diff
Kitchen diner	Far	Speech	No Diff/Diff
Bedroom	Far	Music	No Diff/Refl Diff
Bedroom	Near	Speech	No Diff/Refl Diff

The experiment was run over two sessions to reduce listener fatigue and each session lasted between 30 and 45 min.

### Subjects

In total, 16 people participated in the experiment. Of them, 13 identified as male and three identified as female and, therefore, female listeners are under-represented in this study. The participants were trained listeners consisting of students and researchers from the Institute of Sound Recording, University of Surrey. Two authors of this paper participated in the experiment. No subjects reported abnormal or impaired hearing.

### Results

The mean absolute error ($0.87\pm 0.5$) between repeated cases was largely consistent between participants, so no participants were excluded from the data. For this analysis, the first rating of the repeated cases from each participant was included. Conducting the analysis with the second rating does not change the results that are statistically significant.

**Fig. 5:**
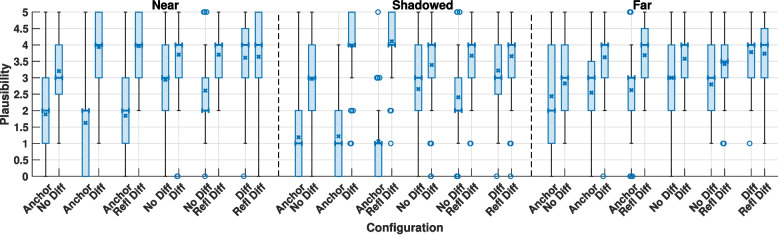
Boxplot showing the median (bold bar), mean (cross), interquartile range (box) and outliers (circle) of paired comparisons rating plausibility between each of the IE model configurations divided by zone for all scenes and programme materials

The plausibility ratings for each paired comparison are shown in Fig. 5 divided by zone. The box plots exhibit large ranges, which reflects the variation in the participants’ internal references. The plots show some variation between identical stimuli, as absolute ratings can vary depending on the stimuli being compared against [49]. Therefore, it can be useful to interpret the results using the relative plausibility between stimuli.

#### Wilcoxon signed rank test

A Shapiro-Wilk test revealed that 23 of 36 results followed a normal distribution. A number of the test cases violate the assumption of normality, therefore a two-sided, paired, nonparametric Wilcoxon signed rank test was used to test the hypothesis of significant differences in plausibility within pairs of acoustic simulations. It was hypothesised that this would also vary between zones. Anchor pairs were excluded from this test. The modified false discovery rate (mFDR) (also known as the B-Y Method) [50] was used to adjust the significance value for multiple comparisons, as it is a less conservative measure than the commonly used Bonferroni correction, but still controls the number of false positives. The results presented in Table 3 show significant differences within the No Diff/Diff and No Diff/Refl Diff pairs in all three zones. A significant difference was found within the Diff/Refl Diff pair only in the shadowed zone.

**Table 3:** Results of the two-sided Wilcoxon-signed rank test comparing the plausibility of three acoustic simulation pairs divided by zone for all scenes and programme materials

Pair	Zone	Sig.
No Diff/Diff	Near	$\boldsymbol{<}$**0.001**
	Shadowed	$\boldsymbol{<}$**0.001**
	Far	$\boldsymbol{<}$**0.001**
No Diff/Refl Diff	Near	$\boldsymbol{<}$**0.001**
	Shadowed	$\boldsymbol{<}$**0.001**
	Far	$\boldsymbol{<}$**0.001**
Diff/Refl Diff	Near	0.719
	Shadowed	**0.004**
	Far	0.518

The significance value is set using the mFDR as 0.017. Bold values indicate statistically significant differences

#### ANOVA

The influence of factors such as reverberation and distance from the edge on the relevance of diffraction modelling also warrants investigation. A four-way ANOVA was performed to examine the effect of room, zone, programme material, and model pair on the difference in plausibility between acoustic simulations. In order to examine the effect of simulations with and without diffraction, only the No Diff/Diff and No Diff/Refl Diff cases were included in this ANOVA analysis. The difference in plausibility between models was calculated for each paired comparison (e.g., $P_{\text {Diff}} - P_{\text {No Diff}}$, where *P* denotes the plausibility rating). Although the ANOVA test assumes that the data is normally distributed, it has demonstrated robustness to Type-I errors even when this assumption is violated [51]. The test showed a statistically significant interaction between scenes (*F*(1, 360) = 4.120, *p *= 0.020), zones (*F*(2, 336) = 3.140, *p *= 0.044), programme materials (*F*(1, 336) = 5.086, *p* = 0.025) and model pairs (*F*(1, 336) = 4.752, *p* = 0.030). This shows that the differences in plausibility between the cases with and without diffraction varied between the different scenes and zones.

**Fig. 6:**
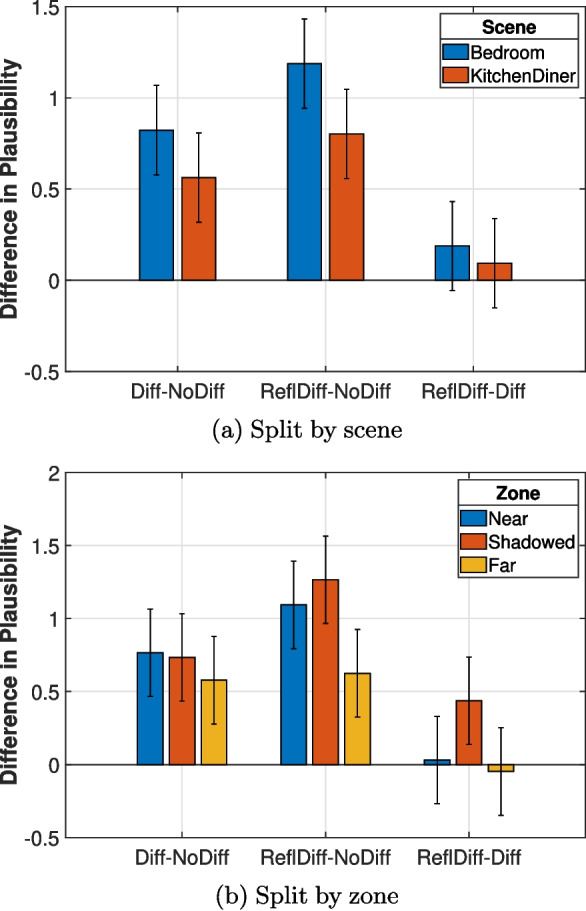
The estimated marginal means of differences in plausibility between each paired comparison for all programme materials. A value greater than zero means, for instance, that Diff is more plausible than No Diff

A second ANOVA analysis was performed that included the Diff/Refl Diff pair. This analysis provided the estimated marginal means for each data group, representing the mean difference in plausibility between two acoustic simulations. The confidence intervals were found using the least squares difference method. These results are shown in Fig. 6, where a value greater than zero indicates a significant difference within the pair. Figure 6a shows that the mean difference in plausibility is reduced in the kitchen diner compared to the bedroom. A similar observation is made between the near and far zones in Fig. 6b. As the receiver moves further from the diffracting edge, the difference in plausibility decreases. Figure 6b indicates that both the Diff and Refl Diff conditions result in an increased plausibility compared to the No Diff condition. Furthermore, it shows that Refl Diff increases the perceived plausibility in the shadowed zone compared to the Diff condition. This finding is consistent with the result of the prior Wilcoxon signed rank test.

#### Participants

Rating plausibility is dependent on participants’ internal references. In some cases, this may be incorrect or a rough approximation of reality. A subject who has spent time with a given task or environment is more likely to have an accurate internal reference as they have spent the time paying attention to the scenario [45]. In some cases, two stimuli were rated similarly plausible but with small, medium or large differences on the second rating screen. This suggests that the participant was unable to distinguish one as more plausible than another despite the audible differences. In other cases, the stimuli were different but similarly implausible. For example, the anchor and no diffraction cases in the shadow zone may both lead to incorrect localisation but in different directions (through the occluding wall and from the opposite reflecting wall, respectively).

**Fig. 7:**
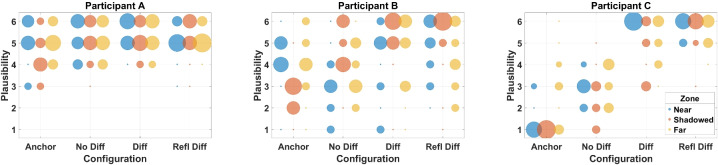
The distribution of plausibility ratings for three participants divided by acoustic simulation and zone for all scenes and programme materials. A large dot size indicates a large number of ratings

This can be represented by plotting representative results from individual participants as shown in Fig. 7. Participant A shows very little variation between models, including the anchor, and does not rate anything as fully implausible. However, their similarity results (not shown here) show variation, and therefore, this suggests either a low threshold for plausibility or a lack of specificity on what is and is not plausible. Participant B reported low plausibility for the anchor case and increased plausibility with the addition of diffraction modelling in the Shadowed Zone, but large variations in the Near and Far Zones. The recorded movement data showed very little exploration within the zones, and therefore, they may have missed useful perceptual information in these zones that occurs when crossing the shadow boundary. Participant C shows a clear trend across all zones and substantially less variation. This suggests a clear internal reference of how the acoustics change for an occluded sound source.

Following the experiment, many participants commented that the perceived location of the occluded sound source affected their plausibility ratings. Hearing the source as from the diffracting edge was perceived as plausible. In the Anchor case or No Diff case, the sound could be localised through the wall or from a nearby reflecting wall, reducing the plausibility. This agrees with previous findings in anechoic environments [33, 34] and suggests that diffraction also affects localisation perception in reverberant conditions.

### Discussion

It is of interest how RE and RT influence the perception of diffraction modelling. The total energy in dB for a given room impulse response (*h*[*n*]) can be calculated as

1 $$\begin{aligned} L = 10\log _{10}\left( \sum \limits _{n = 0}^{N} h[n]^2\right) . \end{aligned}$$

This was calculated for the No Diff case for each zone from 42 listener positions spaced in a 6 by 7 grid with 20 cm spacing. Since the direct sound is identical in both scenes, comparing the total energy between scenes describes changes in RE. This found that the total energy in the kitchen diner was 2.0 dB, 3.8 dB and 2.4 dB higher than the bedroom in the Near, Shadowed and Far Zones, respectively. Given the results shown in Fig. 6a, this may suggest that increasing the RE reduces the difference in plausibility between simulations with and without diffraction. Alternatively, the RT is also higher in the kitchen diner compared to the bedroom, so this may be the cause of the difference between scenes. Disentangling these two parameters is not trivial and requires rooms of varying volumes and absorption parameters. This would also change the early reflection patterns between rooms and is left to future research.

In an indoor environment, as the distance between a source and the listener increases, the direct-to-reverberant ratio will decrease [52]. In the same way, as the listener moves from the Near Zone (0 m to 1.5 m from the diffracting edge) to the Far Zone (1.5 m to 2.7 m from the diffracting edge), the ratio of early (direct and diffracted sound) to late reverberation decreases. A trend is suggested in Fig. 6b that the difference in plausibility decreases as this ratio decreases. This warrants further investigation in larger rooms, where distances between the source, listener and diffracting edge can be greater.

These results demonstrate that diffraction modelling increases the plausibility of an acoustic model in an L-shaped room. They also provide evidence that diffracted reflection paths should be modelled in the shadow region, while previous experiments by Torres et al. [6] were inconclusive. As in Calamia et al. [7], the results from the Near and Far zones show that modelling diffraction across the shadow boundary is important. Including diffraction in the Shadowed Zone also increased the plausibility, which supports their findings that culling paths based on angular distance from the shadow boundary is less effective when sources are occluded [7].

## Experiment 2: similarity

With the knowledge that diffraction modelling increases plausibility under most conditions, it is of interest whether more efficient models can be used in place of BTMS. A second experiment was conducted to assess the plausibility of these models compared to BTMS.

### Subjects

In total, 16 people participated in the experiment. Of them, 13 identified as male and three identified as female and, therefore, female listeners are under-represented in this study. The participants were trained listeners consisting of students and researchers from the Institute of Sound Recording, University of Surrey. One of the subjects was an author of this paper. No subjects reported abnormal or impaired hearing.

### Methodology

An additional outdoor scene was created for this experiment to provide a critical test case with no late reverberation, increasing the audible differences between simulations. In order to reduce the number of test cases, only the Near Zone and Shadow Zone were used as the most critical cases from Experiment 1. The following diffraction models were compared:

BTMS: as described in [18];
NN-IIR: the model 2 kFLOP network denoted NNSmall in [21];
UDFA: single-term approximation for shadow zone only diffraction described in [20] (eq. 7) with four shelving filters;
UTD: 4th-order Linkwitz-Riley filter with cut-off frequencies 176 Hz, 775 Hz and 3408 Hz as described in [53] with the shadow zone interpolation as proposed in [54];
Anchor: 1st-order low-pass filter (LPF) with a cut-off frequency of 1 kHz.

The models (excluding BTMS) only render diffraction in the shadow region, and therefore they enforce a flat frequency response at the shadow boundary. They interpolate between this and the true diffraction frequency response as the bending angle between the source and receiver increases. This guarantees a smooth response across the shadow boundary but lowers the physical accuracy compared to BTMS. The anchor was chosen to approximate the low-pass frequency response of diffraction without any dependence on the relative source and listener positions.

The acoustic model was set to the Refl Diff case from Experiment 1. To limit the number of test pages, each diffraction model was compared with BTMS and the anchor case. The anchor case was the most different from the other models, so only comparing it to BTMS could have made it identifiable as the anchor case due to the limited number of appearances. Comparisons with the other diffraction models prevented this. No direct comparisons were made between UTD, NN-IIR and UDFA.

The experiment consisted of three scenes, two zones, two pieces of program material and seven comparisons (84 pages). Each participant attended two sessions, each taking between 30 and 45 min.

### Results

**Fig. 8:**
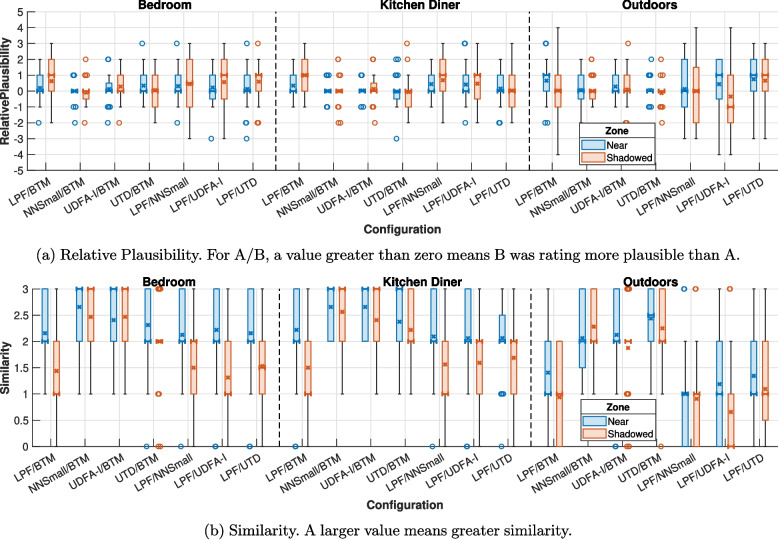
Boxplot showing the median (bold bar), mean (cross), interquartile range (box) and outliers (circle) for the relative plausibility and similarity between diffraction models for each scene split by zone for all programme materials

Figure 8 shows the relative plausibility and similarity ratings of diffraction model pairs in each scene and zone. Table 4 shows that, in most cases, the plausibility of the outdoor scene was rated lower across all models compared to the two indoor scenes, suggesting that the acoustic model was less suited to the sparsity of the outdoor scene. The scene still provides useful results for comparing the different diffraction models.

**Table 4:** The mean plausibility rating of each model in each zone and scene

Model	Scene	Plausibility
BTMS	Bedroom	N: 3.38, S: 3.45
	Kitchen diner	N: 3.51, S: 3.57
	Outdoors	N: 3.27, S: 2.59
NN-IIR	Bedroom	N: 3.44, S: 3.38
	Kitchen diner	N: 3.69, S: 3.55
	Outdoors	N: 2.95, S: 2.56
UDFA	Bedroom	N: 3.30, S: 3.48
	Kitchen diner	N: 3.64, S: 3.58
	Outdoors	N: 3.00, S: 2.25
UTD	Bedroom	N: 3.16, S: 3.36
	Kitchen diner	N: 3.52, S: 3.28
	Outdoors	N: 3.31, S: 2.83
LPF	Bedroom	N: 3.12, S: 2.90
	Kitchen diner	N: 3.30, S: 2.99
	Outdoors	N: 2.54, S: 2.34

N is the Near Zone and S is the Shadowed Zone. This was calculated from every rating of each model independent of the paired comparison or programme material

#### Wilcoxon signed rank test

**Fig. 9:**
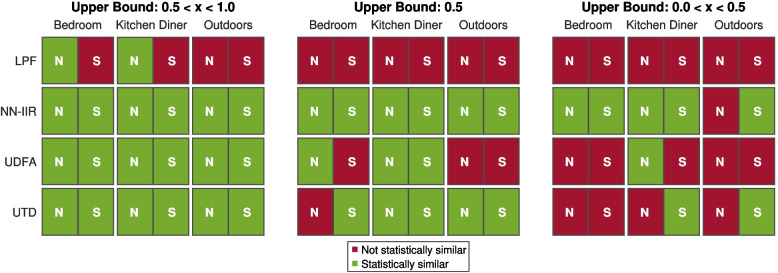
Equivalence tests divided by scene and zone for each of the efficient diffraction models compared to BTMS for all programme materials. N is the Near Zone and S is the Shadowed Zone. Green means that they can be considered equivalent to BTMS within the given upper bound

A Kolmogorov-Smirnov test found that all of the results had a statistically significant deviation from a normal distribution, and therefore, a Wilcoxon signed rank test was used for analysis. Typically, paired comparisons are tested for differences. However, failing to find a statistically significant difference between two models does not allow for the conclusion that two models are similar. Instead, an equivalence test [55] can be used. This rejects differences larger than the chosen upper and lower bounds, allowing the conclusion that the difference between two models is close enough to zero to be insignificant. In this case, an upper bound is sufficient as an efficient model that is either similarly plausible or more plausible than BTMS can be considered successful. A one-sided, paired, nonparametric Wilcoxon signed rank test was used to test the hypothesis that the differences in plausibility between NN-IIR, UDFA UTD or LPF and BTMS are less than a given upper bound. There is no obvious choice for an upper bound in this case; however, since the Wilcoxon test is a rank-based test and the ratings were obtained on an integer scale, the test result changes at 0.5 increments. Therefore, to provide the most information to the reader, the results shown in Fig. 9 represent three tests with upper bounds of 0.5 < x < 1.0, 0.5 and 0 < x < 0.5, respectively.

Within each of the tests, the mFDR (also known as the B-Y Method) [50] was used to adjust the significance value for multiple comparisons.

### Discussion

These results suggest that efficient diffraction models can be used in place of BTMS with limited reductions in plausibility. It can be seen in Table 4 that NN-IIR, UDFA and UTD were rated more plausible than the simple LPF model in most cases. Although no statistically significant differences were found between the models, at the highest upper bound (0.5 < x < 1.0) the LPF model was statistically similar to BTMS for only 2 of 6 cases. This indicates that an accurate frequency response is important for plausible diffraction modelling and is consistent with previous findings by Mannall et al. [9]. The outdoor scene seems to have been the most critical, suggesting it is a useful test case for future evaluations of new diffraction models. In general, Fig. 9 shows that efficient models were rated similar to BTMS in the kitchen diner for lower upper bounds than in the bedroom, suggesting that increasing the level of reverberation makes differentiation between models more difficult.

At the lowest upper bound (0 < x < 0.5), the NN-IIR model was rated equivalent to BTMS in 5 of 6 cases. Figure 9 shows that the UTD model was rated equivalent to BTMS in the outdoor scene at lower thresholds than in the bedroom scene. UTD assumes that all edges are infinitely long, leading to low-frequency errors with short edges, but in the outdoor scene, the edges are generally longer, so this assumption is less problematic. The NN-IIR model was found to be perceptually similar to BTMS in more cases than UDFA and UTD while also using a more efficient IIR filter structure [21]. The cost of calculating target filter coefficients for NN-IIR is larger than for UDFA [24].

Despite UDFA having similar or smaller frequency domain error compared to BTMS as NN-IIR, it performed slightly worse in this experiment. NN-IIR and UTD use the same shadow boundary interpolation method [54], while the single-term UDFA approximation uses a different approach [20]. Neither interpolation method is physically accurate, but it may be that the UDFA method is less perceptually plausible.

## Conclusion

This paper presented two experiments on the perception of diffraction in L-shaped rooms. The results of the first experiment found that including diffraction modelling in the acoustic simulation led to a significant increase in the perceived plausibility of the virtual environment. It is particularly noteworthy that significant increases in plausibility were found when diffracted reflection paths were modelled. Current real-time acoustic modelling often neglects these paths [15, 56], but the results here demonstrate the benefits of including them in the shadow region. The experiment found significant differences between rooms with differing REs and between different locations with the rooms. This suggests that increasing RE or the distance between the listener and the diffracting edge decreases the perceptual importance of diffraction modelling.

The second experiment showed that the NN-IIR model [21] was similarly plausible to the physically accurate BTMS model in 5 out of 6 cases with the lowest upper bound. This compares to 1 out of 6 for the UDFA model and 2 out of 6 for the UTD model [53]. At the highest upper bound, all three models were found to be similar to BTMS and outperformed the naive LPF case. Scenes with lower levels of reverberation were the most critical when evaluating differences between models. Using an efficient IIR filter model can lead to significant performance increases compared to BTMS, especially when multiple diffracting paths are being modelled.

Future research could aim to locate RE and distance thresholds for the inclusion of diffraction, including outside the shadow region. These findings could be applied to path-finding algorithms to prune edges or regions within a room. Further experiments could investigate perception within more complex environments that include heavy occlusion and multiple significant diffracting paths. Higher-order diffraction would also be of interest as only 1st-order diffraction was considered here. This poses a particular challenge for efficient diffraction models [21, 24] but has not been evaluated through perceptual experiments.

## Data Availability

The RoomAcoustiC++ library used in the experiments is openly available on GitHub: https://github.com/jmannall/RoomAcoustiCpp. Data sharing is not applicable to this article as no datasets were generated or analysed during the current study.

## Footnotes

1 https://github.com/jmannall/RoomAcoustiCpp
